# Power-Law Modeling of Cancer Cell Fates Driven by Signaling Data to Reveal Drug Effects

**DOI:** 10.1371/journal.pone.0165049

**Published:** 2016-10-20

**Authors:** Fan Zhang, Min Wu, Chee Keong Kwoh, Jie Zheng

**Affiliations:** 1 Biomedical Informatics Lab, School of Computer Science and Engineering, Nanyang Technological University, 639798, Singapore, Singapore; 2 Data Analytic Department, Institute for Infocomm Research, Agency for Science, Technology and Research, 138632, Singapore, Singapore; 3 Complexity Institute, Nanyang Technological University, 637723, Singapore, Singapore; 4 Genome Institute of Singapore, Agency for Science, Technology and Research, 138672, Singapore, Singapore; Friedrich-Alexander-Universitat Erlangen-Nurnberg, GERMANY

## Abstract

Extracellular signals are captured and transmitted by signaling proteins inside a cell. An important type of cellular responses to the signals is the cell fate decision, *e.g.*, apoptosis. However, the underlying mechanisms of cell fate regulation are still unclear, thus comprehensive and detailed kinetic models are not yet available. Alternatively, data-driven models are promising to bridge signaling data with the phenotypic measurements of cell fates. The traditional linear model for data-driven modeling of signaling pathways has its limitations because it assumes that the a cell fate is proportional to the activities of signaling proteins, which is unlikely in the complex biological systems. Therefore, we propose a power-law model to relate the activities of all the measured signaling proteins to the probabilities of cell fates. In our experiments, we compared our nonlinear power-law model with the linear model on three cancer datasets with phosphoproteomics and cell fate measurements, which demonstrated that the nonlinear model has superior performance on cell fates prediction. By *in silico* simulation of virtual protein knock-down, the proposed model is able to reveal drug effects which can complement traditional approaches such as binding affinity analysis. Moreover, our model is able to capture cell line specific information to distinguish one cell line from another in cell fate prediction. Our results show that the power-law data-driven model is able to perform better in cell fate prediction and provide more insights into the signaling pathways for cancer cell fates than the linear model.

## Introduction

The extracellular signals are captured and transmitted into the cells through signaling pathways. Generally, signal transduction involves various protein modifications such as phosphorylation. The signals are transmitted down to the nucleus or other cellular organelles to regulate their physiological functions and control the cell fates (*e.g.*, apoptosis, proliferation and cell cycle). Although wet-lab experimental evidence has suggested some proteins to be crucial to one of the cell fates (*e.g.*, engagement of TNF is able to trigger cell death [1]), it is still unclear how the signaling proteins are integrated to systematically decide the cell fates. Therefore, computational models are needed to study how the signaling “input” is related to the cell fate “output”.

Many statistical methods have been employed to model the relationship between the input and the output of a system, including the autoregressive with exogenous terms (ARX) model and the power-law statistical model. In an ARX model [2, 3] the current value of each variable of interest is predicted based on its previous values in the time series and the past values of the exogenous series. The power-law distributions [4, 5], in which some quantity varies as a power of another quantity, have been investigated and applied in an extraordinarily diverse range of scientific areas, such as earth and planetary sciences [6] and social sciences [7]. In biology, studies have also shown that many complex phenomena of living systems scale with the biomass in a simple power-law fashion. For example, the metabolic rate scales as the $\frac{3}{4}$ power of mass [8] across nearly all life forms and the population density is inversely proportional to the individual body size with a power $-\frac{3}{4}$ [8]. However, to the best of our knowledge the power law has not been applied to studying the specific relationship between cellular signaling and cell fates (*e.g.*, apoptosis of cancer cells).

A straightforward way to model a signaling pathway is to assume a linear relationship between the inputs and the outputs. Janes *et al.* [9], Gaudet *et al.* [10] and Lee *et al.* [11] applied linear models to identify the effects of extracellular perturbations such as drug combinations on cancer cell death. They assumed that the phenotypic outputs are linearly related to the signaling inputs. By using the method of partial least squares regression (PLSR), different cellular responses between cell lines as well as various drug treatments were detected. However, signaling pathways are so complex in their interactions (*e.g.*, multiple levels of cross-talks and feedbacks) that their behaviours are expected to be nonlinear [12, 13]. Legewie *et al.* [14] found that the inhibition of pathways that control apoptosis results in a positive feedback leading to bistability. Eissing *et al.* [15] also revealed that a positive feedback loop plays an important role in signal-induced apoptosis. Callard *et al.* [16] summarized several nonlinear properties of biological systems, such as the difficulty in predicting the behaviors of the whole system based on parts, a small change in a specific component can have significant effect on the system thus making this component essential. They also studied typical cytokine dose-response curves and indicated that cells have no response if the concentration of cytokine is below a certain value; on the other hand, the cellular response is approximately an exponential function of the signal quantity until the response reaches a plateau maximum.

In this paper, we propose a power-law model to predict the probabilities of cell fates based on the activity levels (*e.g.*, the phosphorylation levels) of signaling proteins. Simulations based on generalized Boolean network and ordinary differential equations are exploited for model validation. The results show that the proposed model has better performance than the linear models on cell fate prediction. Moreover, the proposed model is also able to identify the signal transduction events that are blocked by the drugs, thereby revealing the drug-induced signaling pathway alterations. When our nonlinear model is applied to cell line discrimination, the cell lines are much better separated and more concentrated compared with the results of the linear model. Testing on 3 different cancer datasets [10, 11, 17] suggests that our nonlinear power-law model has some superiority over the linear model.

## Methods and Data

### Data

We downloaded 3 cancer datasets to evaluate the performance of the proposed model. The first dataset is from the published work of Lee *et al.* [11]. In this dataset, activity levels of 32 signaling proteins are measured at 8 time points. The measurements of 6 types of cell fates (*i.e.* apoptosis, proliferation and cell cycle phases including G1, S, G2 and M) refer to the normalized numbers of cells of the cell fates which are measured at 5 different time points using flow cytometry. 6 treatments designed for 3 breast cancer cell lines (*i.e.* BT20, MDA-MB-453 and MCF7) are performed on triplicate plates (*i.e.* 3 replicates). In each cell line, there are 18 observations of both signals and cell fates at each time point. In total, there are 432 (3 replicates × 8 time points × 6 treatments × 3 cell lines) measurements for each signaling protein and 270 (3 replicates × 5 time points × 6 treatments × 3 cell lines) observations for each cell fate. The preprocessing of the data was as follows. First, we extracted the signaling “inputs” and cell fate “outputs” with the same experimental treatments and the cell lines. Second, we removed the observations with 0 or negative values or NANs (not measured). Finally, there are 164 observations left (75 of BT20, 56 of MDA-MB-453 and 33 of MCF7). We further calculated the proportion of apoptotic cells under each observation, because our model aims to predict the cell apoptosis.

The second dataset [10] contains measurements of phosphorylation levels acquired from HT-29 cells (human colorectal adenocarcinoma cell line) under 12 different treatments (*i.e.* combination of 3 stimuli of TNFR, EGFR and Insulin). In each treatment, the phosphorylation levels of 19 proteins are measured at 13 time points. Four types of cell apoptotic responses (*i.e.* cleaved caspase-3, sub-G1, phosphatidylserine exposure and membrane permeabilization) are measured at 3 time points. For every experiment there are 3 replicates (except at time point 0 at which 6 replicates were made). As such, the second dataset consists of 504 (3 replicates × (13+1) time points × 12 treatments) measurements for each signaling protein and 108 (3 replicates × 3 time points × 12 treatments) measurements for each cell response.

The third dataset is from the DREAM8 (Dialogue for Reverse Engineering Assessments and Methods) challenge in 2013 [17]. The DREAM8 dataset consists of 4 breast cancer cell lines, namely, BT20, BT549, MCF7 and UACC812, and each cell line is perturbed by the combination of 3 inhibitors and 8 ligand stimuli. These 3 inhibitors target at AKT, AKT with MEK, and FGFR1 with FGFR3, respectively. The 8 ligand stimuli include Serum, PBS, EGF, Insulin, FGF1, HGF, NRG1 and IGF1. In each cell line, a number of phosphoproteins are measured at 7 time points after a perturbation, *e.g.*, 48 phosphoproteins in BT20, 45 in BT549, 41 in MCF7 and 45 in UACC812. Overall, there are 234, 252, 312 and 312 observations for cell lines BT20, BT549, MCF7 and UACC812, respectively. Note that this dataset contains only breast cancer proteomics data without any measurement of cellular response.

### Nonlinear power-law model

Suppose *P* is the score indicate the probability of cell death, *x*_*i*_ (*i* = 1…*n*, where *n* is the number of measured signaling proteins) indicates the signaling activity (*e.g.*, phosphorylation level) of the *i*-th protein and *α*_*i*_ (*i* = 1…*n*) represents the contribution of the *i*-th protein to the cell death. Then our good is to construct a statistical model $P=F(x_{1},x_{2},\ldots ,x_{n})$ that relates the signaling “inputs” to the phenotypic “outputs”. The most commonly used linear model [10, 11] can be written as

$$\begin{array}{c} P=\sum_{i=1}^{n}\alpha_{i}x_{i}+\varepsilon , \end{array}$$ (1)

where *ε* is a noise term representing the influence of the unmeasured signaling proteins to cell death.

If we regard the activities of signaling proteins as the independent variables and the cell death as the dependent variable, the coefficient *α*_*i*_ can be identified by regression methods. These parameters can be used to capture the covariance between independent and dependent variables, *i.e.*, to infer their causal relationship [18].

**Fig 1:**
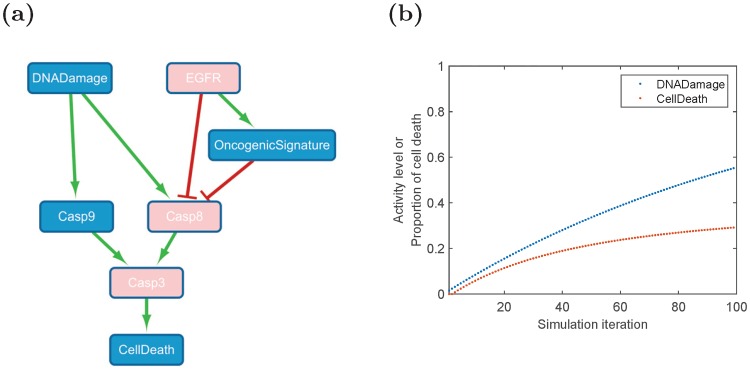
(a) A signaling network for cell death regulation [11]. Cell death is enhanced due to DNA damage while inhibited by the signals transmitted from EGFR. The arrow shape represents activation while a flat-head edge means inhibition. Pink nodes denote the species that have experimental measurements. (b) The simulation results of DNA Damage and Cell Death.

Despite promising results of using the linear model for signaling analysis [11], it is unlikely that the probabilities of cell fates are proportional to every molecular species with a constant ratio, *i.e.*, the linear function in Eq (1) may be unable to model the nonlinear relationship in a biological system. To search for suitable nonlinear models, we first designed simulation experiments to study how cell fates are related to signaling proteins one at a time, *i.e.*, construction of a function with a single variable. We first extracted a network (Fig 1(a)), which describes cell death regulation, from the model suggested by Lee *et al.* [11]. In this network, cell death is enhanced by DNA damage while inhibited by EGFR. Then SimBoolNet, an open source Cytoscape plugin which had been designed for dynamically simulating the process of signal transduction based on an extended Boolean network model [19], was employed to run the simulations. We selected EGFR and DNA Damage as the input signals. Since we have insufficient prior knowledge to determine the parameters here, the default settings of the software were employed: the edge weights (value ∈[0, 1]) of both activation and blockage were set to 0.8; moreover, the input levels (value ∈[0, 1]) of EGFR and DNA Damage were both set to 0.8. The simulation generated time-series data containing 100 time points. Fig 1(b) shows the simulation results of DNA Damage and Cell Death. Considering the simulated time-series data as vectors with length of 100, the Curve Fitting Tool from MATLAB R2015b was used to analyze the data and identify the function that best describes the relationship between cell death and each node in the network. The results may provide a clue to help construct a function that relates cell death with all signaling proteins (*i.e.* a multivariate function).

For comparison, we employed four different models, *i.e.*, power function, linear function, exponential function and Gaussian function (Eq 2), to do the regression. In Eq 2, *y* and *x* represent the cell death and the activity level of a signaling protein at the same time point *t* (*t* = 1…100). Four statistical measures generated by the Curve Fitting Tool were used to evaluate the goodness of fit: sum of squares due to error (SSE), R-Square (value ∈[0, 1]), degrees of freedom adjusted R-Square (value < = 1) and root mean squared error (RMSE). For SSE and RMSE whose values are always between 0 and 1, a value closer to 0 indicates that the model has a smaller random error component and that the fit is better. By contrast, for R-Square and adjusted R-Square, a value closer to 1 indicates a better fit.

$$\begin{array}{ll} Power: & y=a\times x^{b}\\ Linear: & y=a\times x+b\\ Exponential: & y=a\times exp^{b\times x}\\ Gaussian: & y=a\times exp^{-\frac{{\left(x-b\right)}^{2}}{c^{2}}} \end{array}$$ (2)

To relate a dependent variable to more than one independent variables in a biological process, Savageau used the *Power-Law* formalism in biochemical systems theory such that one variable (*e.g.*, the rate of a biological process) is represented as a power function of others (*e.g.*, the concentrations of biological moleculars) [20, 21]. Since many biological processes can be approximated by a straight line in a *log-log* plot over a wide range [22], we formulated the model as a product of power functions of the variables, given as

$$\begin{array}{c} y=c\cdot \prod_{i=1}^{n}x_{i}^{\beta_{i}}, \end{array}$$ (3)

where *y* and *x*_*i*_ represent the components of the biochemical system, and *c* is a constant.

Therefore, we combined all the 6 nodes (*i.e.*, DNA Damage, EGFR, Oncogenic Signature, Casp8, Casp9 and Casp3) together as independent variables and fit the simulation data into Eqs (1) and (3), respectively. Least-squares regression was employed to estimate the coefficients of the regression functions, and root mean squared error (RMSE) was used to measure the goodness of fit.

Given the results in Section “Analysis of simulation data”, we propose a model to relate the cell death to the activities of all the measured signaling proteins, given as

$$\begin{array}{c} P=e^{\beta_{0}}\cdot \prod_{i=1}^{n}x_{i}^{\beta_{i}}+\varepsilon , \end{array}$$ (4)

where the parameter *β*_*i*_(*i* = 1…*n*) represents the contribution of the *i*th protein to the cell death and *ε* is a small constant term to keep the function from crossing the origin (*i.e.*, the value of a dependent variable is not required to be zero when the value of an independent variable equals zero). The proposed model is used to describe the system at any time point during a biological process, not necessarily the steady state.

### CellDesigner simulation

In addition to the above simulation using Boolean Network (*i.e.*, SimBoolNet), we also compared the linear model with the nonlinear model on simulating data generated by solving ordinary differential equations (ODEs) which is a continuous model of biological systems with known biochemical kinetics. ODE models have been successfully applied to modeling numerous processes in living systems [23–26]. CellDesigner is a process diagram editor for biochemical networks as well as an ODE-based simulator [27]. We chose CellDesigher to test if our nonlinear model can accurately describe the relationships generated with an ODE-based model.

We selected from the BioModels Database [28] an ODE-based model that was constructed to quantitatively analyze the pathways responsible for controlling extrinsic apoptosis in single cells [9]. The network is composed of 112 nodes. Simulations were conducted using the simulator of CellDesigner with the default setting of the parameters, which spanned 20,000 time points. We then selected data every 200 time points and collected 100 observations. Before fitting the simulation data, the molecular species whose concentrations remained 0 during all the time points of the simulation were removed and as such 58 nodes were retained. Apoptosis was chosen as the dependent variable and all the other 57 nodes were regarded as the independent variables. PLSR was employed to preform the regression. Five-fold cross-validation was carried out, and the parameters in Eqs (1) and (4) were learned based on the training data set. The prediction was performed on the testing data set. The above process was repeated for 200 times to generate 1000 outputs.

Both the Spearman and Pearson correlation coefficients between the model predictions and the testing data were calculated to assess the performances of Eqs (1) and (4). The Spearman correlation coefficient benchmarks monotonic relationship and the Pearson correlation coefficient is a measure of the linear correlation between two variables. If both coefficients are high (*e.g.*, higher than 0.9), the model predictions are considered to have high accuracy.

### Evaluation metrics of model performance

In addition to simulation studies, we also carried out cross-validation on the 3 real cancer datasets and employed PLSR to estimate the parameters. PLSR is used when the goal is to predict causal relations between independent and dependent variables. It seeks to maximize the correlations between the principle components of independent variables and those of dependent variables. Therefore, PLSR emphasizes the independent variables that have strong covariance with the dependent variables.

For the nonlinear model, we substituted the cell death data and signaling protein data of the training data set into Eq (5) which is the logarithmic deformation of Eq (4), given as

$$\begin{array}{c} \ln\left(P-\varepsilon \right)=\beta_{0}+\sum_{i=1}^{n}\beta_{i}\ln\left(x_{i}\right), \end{array}$$ (5)

and employed PLSR to estimate the parameter *β*_*i*_ (where *i* = 0…*n*, and *n* is the total number of measured signaling proteins). For the linear model, we directly use the original PLSR to estimate all the parameters from the training data set. Hence, we were able to make predictions using both the linear and the nonlinear models on the testing data set.

Besides the Spearman correlation and the Pearson correlation, Kullback-Leibler divergence was employed as the loss function to assess the discrepancy between the predicted and the actual probabilities (*e.g.*, the proportion of dead cells among all the cells in the real data is considered as the cell death score). In information theory, the Kullback-Leibler divergence is used to quantify the difference between two distributions [29]. Suppose $\hat{p}$ is the predicted distribution and *p* is the actual distribution, the loss function is written as

$$\begin{array}{c} L\left(p,\hat{p}\right)=\sum_{i=1}^{m}p_{i}\log_{2}\left(p_{i}/{\hat{p}}_{i}\right), \end{array}$$ (6)

where *m* is the total number of predictions. The value of Kullback-Leibler divergence is non-negative. If the distributions of predicted and actual probabilities are perfectly matched, this value will be zero.

### Identification of drug effects

The identification of drug effects plays an important role in biomedical research and pharmaceutical applications. One primary objective is to selectively target a signaling pathway while making the others unaffected. The *in vivo* drug effects on the signaling pathways can be estimated according to the change of the signals downstream the drug target [30]. For example, in Fig 1, if Casp9 is the drug target, the activity of Casp3 will decrease (since the signal transduction from Casp9 to Casp3 is blocked) even though the signal transduction to Casp9 is not blocked.

According to the different treatments, we divided the real data set [11] into two groups: control group and drugged group. Using the control data, we learned the parameters in Eqs (4) and (7). Eq (7) describes the relationship between one signaling protein with all the other measured proteins. The parameters in Eq (7) form a matrix *M* with each element *λ*_*ij*_ representing the influence of the *j*-th signaling protein on the *i*-th protein [31].

$$\begin{array}{c} x_{i}=e^{\lambda_{i0}}\cdot \prod_{j=1}^{n}x_{j}^{\lambda_{ij}}+\varepsilon_{i}, \end{array}$$ (7)

where *λ*_*ij*_ = 0, if *i* = *j*, otherwise *λ*_*ij*_ = 0 will be learnt from the data.

We iteratively selected the *k*-th signaling protein as the blocked protein. Every entry of the *k*-th column of the matrix *M* is thus set to zero, meaning there is no influence of this protein on the other proteins any more. And *β*_*k*_ in Eq (4) is also set to zero to remove the contribution of the *k*-th protein to cell death. Subsequently, the new parameter $\beta_{j}^{\prime }\left(j=1\ldots n,\text{and if }j=k,\beta_{j}^{\prime }=0\right)$ is calculated as $\beta_{j}^{\prime }=\sum_{i=1}^{n}\lambda_{ij}\beta_{i}$ to modify the influence of the *j*-th protein to the cell death after the knock-down of the *k*-th protein. The term *λ*_*ij*_ *β*_*i*_ denotes that the contribution of the *j*-th protein to cell death is made by influencing the activity of the *i*-th protein as an intermediary. Then using the signaling data of the drugged group, the predicted probabilities of cell death were calculated and root mean squared error (RMSE) was employed to measure the goodness of fit to the real data. If the fit is good, it can be inferred that the drug blocks the signal transduction from the drug-targeted nodes to the *k*-th signaling protein. This *in silico* simulation of protein knock-down is thus able to predict drug effects.

## Results and Discussion

### Analysis of simulation data

#### Single variable scenario

Table 1 shows the curve fitting results when the cell death is one of the following functions of EGFR (or DNA Damage), namely power function, linear function, exponential function and Gaussian function, respectively. We can see from the table that the power function has the best fit to the simulation data. Taking EGFR as an example, the SSE and the RMSE of the power function are closest to 0 and the R-Square and the adjusted R-Square of the power function are closest to 1. This means that using a power function is probably the best way to describe the relationship between cell death and EGFR among the four functions. A similar conclusion can be drawn for Oncogenic Signature, Casp8, Casp9 and Casp3 (data not shown). We then tried many other settings (*e.g.*, different edge weights and input levels) to run SimBoolNet which showed that the power function was also the best fit to the simulation data (data not shown).

**Table 1:** Curve fitting results of Power function, Linear function, Gaussian function and Exponential function. The statistics are about EGFR and DNA Damage.

Node	Function	SSE	R-Square	Adjusted R-Square	RMSE
EGFR	Power	1.3 ⋅ 10^−3^	0.99	0.99	3.6 ⋅ 10^−3^
	Linear	2.1 ⋅ 10^−2^	0.97	0.97	1.5 ⋅ 10^−2^
	Exponential	8.0 ⋅ 10^−2^	0.88	0.88	2.9 ⋅ 10^−2^
	Gaussian	1.5 ⋅ 10^−2^	0.98	0.97	1.2 ⋅ 10^−2^
DNA Damage	Power	1.3 ⋅ 10^−3^	0.99	0.99	3.7 ⋅ 10^−3^
	Linear	2.0 ⋅ 10^−2^	0.96	0.96	1.4 ⋅ 10^−2^
	Exponential	7.9 ⋅ 10^−2^	0.88	0.87	2.8 ⋅ 10^−2^
	Gaussian	1.4 ⋅ 10^−2^	0.97	0.97	1.2 ⋅ 10^−2^

#### Multiple variables scenario

Then all the 6 nodes (*i.e.*, DNA Damage, EGFR, Oncogenic Signature, Casp8, Casp9 and Casp3) were combined together as independent variables and fit the simulation data into Eqs (1) and (3). The RMSE values are 6.19 × 10^−5^ and 4.13 × 10^−5^ when the simulation data was fitted into Eqs (1) and (3), respectively. The Pearson’s correlations coefficients between the model predictions and the synthetic data were also calculated. The correlation coefficient between our model predictions and the synthetic data was 0.89, while the coefficient was 0.74 for the linear model prediction. The result shows that Eq (3) fits the simulation data better than Eq (1).

#### CellDesigner simulation

**Fig 2:**
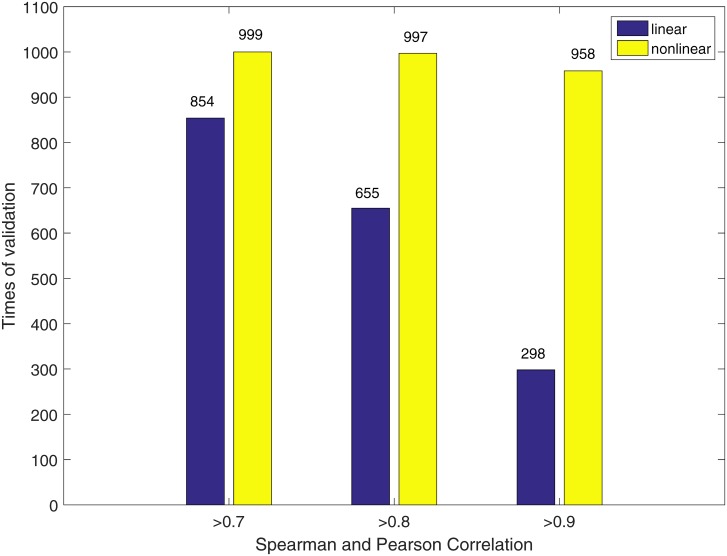
Cross-validation of the linear and the nonlinear models on synthetic data. Five-fold cross-validation of the linear (blue bars) and the nonlinear (yellow bars) models on the simulation data generated with an ODE-based system. For example, 999 out of the total 1000 predictions made by the nonlinear model have both the Spearman and the Pearson correlations with the testing data higher than 0.7.

From Fig 2, it is clear that the number of outputs with both coefficients higher than a threshold (*e.g.*, 0.9, 0.8 and 0.7) from the nonlinear model is larger than that from the linear model. As the threshold increases from 0.7 to 0.9, the superiority of the nonlinear model over the linear model becomes more obvious. The regression results demonstrate that the present nonlinear model fits the ODE-based system simulation data better than the linear model.

### Results on breast cancer cell lines

#### Prediction of cell fate

Based on the phosphorylation levels of 32 signaling proteins at a time point, we intended to predict the cell death at the corresponding time point. Both the linear (Eq (1)) and our nonlinear (time point. Both the linear Eq (4)) models were employed to relate the cell death to the activities of proteins. Cross-validation was used to estimate the performance of both models on cell death prediction.

**Fig 3:**
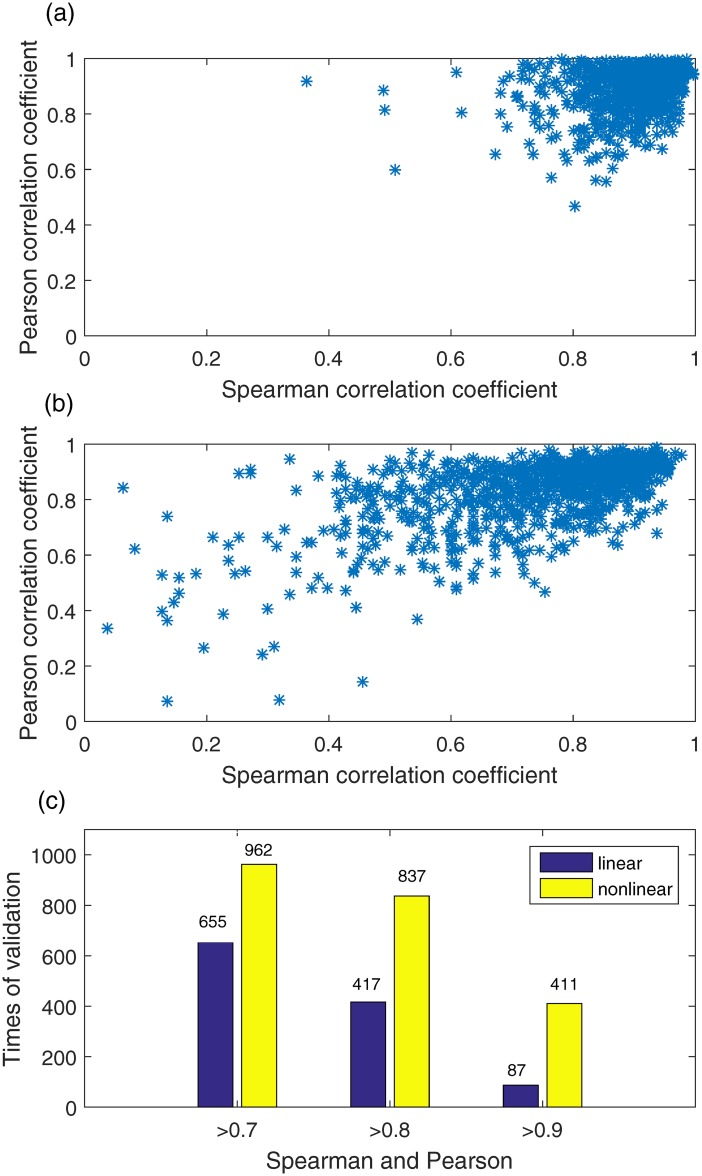
Performance comparison of cell death prediction within each cell line. More outputs have high coefficients in the nonlinear model (a) than in the linear model (b). (c) Comparison between the two models by counting how many outputs have both coefficients higher than a threshold (e.g., 0.7, 0.8 and 0.9).

First we carried out 5-fold cross-validation on the data set within the same cell line. Because the number of observations of cell line MCF7 is smaller than the number of parameters, we ignored this cell line to avoid overfitting. Altogether, we did 100 times of validations and generated 1000 outputs for the linear model and 1000 outputs for the nonlinear model (Fig 3).

Fig 3(a) and 3(b) show that the distribution of the outputs generated by the nonlinear model is more concentrated than the outputs of the linear model in the area where both the Spearman correlation coefficients and the Pearson correlation coefficients are high. Also, it is clear that the number of outputs with both coefficients higher than a threshold (*e.g.*, 0.9, 0.8 and 0.7) from the nonlinear model is larger than that from the linear model (Fig 3(c)). When the threshold is set to 0.7, satisfactory results of the nonlinear model is 96.2%, compared with 65.5% from the linear model. The difference increases from 30.7% to 42.0% when the threshold is 0.8. A remarkable difference (32.4%) is also observed if the threshold is set to 0.9. Moreover, one-way analysis of variance (one-way ANOVA) was used to compare the correlations of model predictions with the real data of cell fate over time points between our model and the linear model. Table 2 shows the means and standard deviations of the correlation coefficients, and the p-value of the one-way ANOVA analysis. We observed that the correlations between our model predictions and the real data are significantly better than the linear model, suggesting that the nonlinear model performs better than the liner model of cell fate prediction with statistical significance.

**Table 2:** The mean and standard deviation of the correlation between model predictions and the real data. The *p*-value of one-way analysis of variance shows that the correlations between our model predictions and the real data are significantly better than the correlations between the linear model predictions and the real data.

	Our model		The linear model		*p*-value from one-way ANOVA
	Mean	SD	Mean	SD	
Spearman correlations	0.90	0.06	0.74	0.17	2.6E-145
Pearson correlations	0.89	0.08	0.82	0.13	2.3E-41

Among 1000 outputs, both the linear and the nonlinear models make 13,100 predictions. Using Eq (6), we calculated Kullback-Leibler divergence for the predictions of both models. 67.2 is obtained by the nonlinear model which is smaller than 277.7 by the linear model. This further demonstrates that the probabilities predicted by our nonlinear model are more accurate.

We then combined 164 observations from all the 3 cell lines as a whole dataset to do 5-fold cross-validation. This experiment was designed for the situation when the data have no cell line information. Cross-validation was run for 200 times and 1000 outputs were generated.

**Fig 4:**
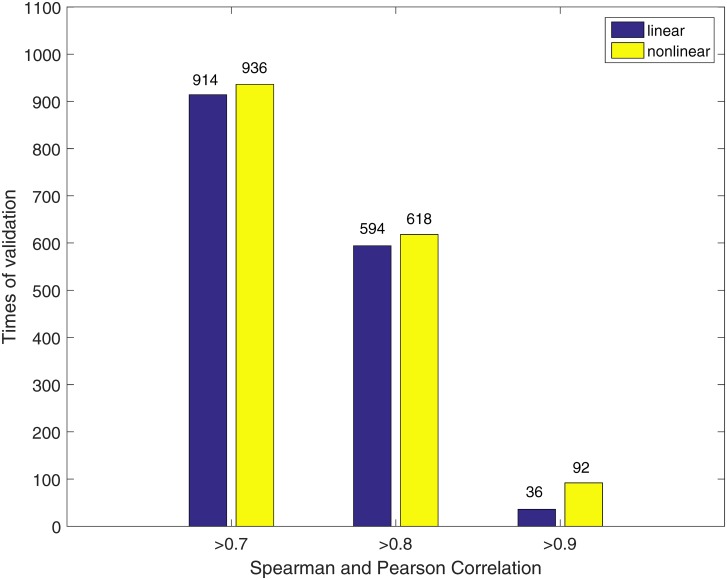
Comparison of cell death prediction by the linear and the nonlinear models when combining all three cell lines together.

Fig 4 displays the performance comparison between the linear and the nonlinear models. It is clear that for all the 3 thresholds, the proposed nonlinear model has superiority over the linear model on cell death prediction. A consistent conclusion can be drawn from the Kullback-Leibler divergence, since for the 32,800 predictions of the linear and the nonlinear models the values are 1,117.5 and 450.5, respectively. Moreover, Akaike information criterion (AIC) was calculated based on Eq (8) where $L(p,\hat{p})$ is the loss function from Eq (6), *k* is the number of parameters and *m* is the number of predictions. Since the numbers of parameters in the linear and the power-law models are the same (*i.e.*, 33), the values of AIC for the linear model and nonlinear model are 10.1 and 8.8, respectively, demonstrating that it has a better fit than the linear model has a better fit.

$$\begin{array}{c} AIC=\ln\left(L\left(p,\hat{p}\right)\right)+\frac{2k}{m}, \end{array}$$ (8)

Then, we tested both the linear and our nonlinear models by training and predicting in alternative cell lines to see which model can perform better. We chose the cell lines of BT20 and MDA-MB-453 but ignored MCF7 because the number of observations in MCF7 is smaller than the number of parameters. Table 3 shows the result of the prediction across cell lines. With regard to the absolute value of the correlation coefficient, the closer it is to 1, the better is the correlation between the predicted and real data. When BT20 is used for training and MDA-MB-453 is used for testing, the nonlinear predictions are more monotonically correlated with the testing data and have less loss of information than the linear predictions, but the linear predictions have better linear correlation with the real data. Alternatively, when MDA-MB-453 is employed as the training data set, the proposed nonlinear model outperforms the linear model on both correlations. Also, the Kullback-Leibler divergence indicates that the nonlinear model can capture more information.

**Table 3:** Performance of the linear and the nonlinear models in predicting cell fate across cell lines.

Train	Test	Model	Spearman	Pearson	Kullback
BT20	MDA	nonlinear	0.64	0.53	0.19
		linear	0.60	0.68	0.23
MDA	BT20	nonlinear	0.70	0.57	0.30
		linear	-0.36	-0.25	1.0

Overall, when predicting cell death probabilities from the activities of signaling proteins, our nonlinear model has some superiority over the linear model.

#### Drug effects identification by *in silico* simulation of protein knock-down

Suppose node *u* is the upstream signaling protein of node *v* and node *u* is a drug target. As such, the drug will block the signal flow from *u* to *v*. The signals transmitted to *v* are decreased while those transmitted to *u* should remain unchanged. The interactions between *v* and other proteins are thus weakened. In the data-driven methods, the correlations between *v* and other nodes in the network (including other proteins and the cell death) are reduced. However, this does not necessarily happen to *u*. Hence, in the dataset of drug treatment, the contributions of *v* to other proteins should be less significant than in the control data, and the impact of the knock-down of *v* on cell responses should be relatively small. Therefore, by setting both *β*_*v*_ in Eq (4) and *λ*_*iv*_ in Eq (7) to zero (*i.e.*, remove the influences of *v* on the other molecules), the predicted probabilities of cell death should have smaller discrepancies (*e.g.*, smaller RMSE) with the real data in the drugged group.

**Fig 5:**
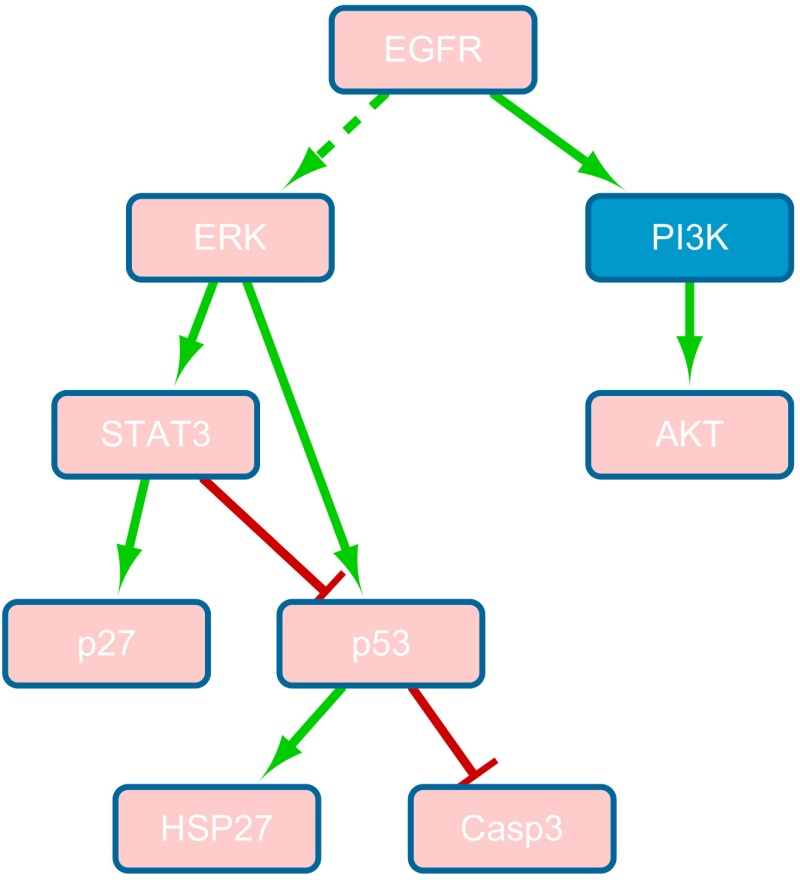
Pathway information extracted from GeneGO database [32]. An arrow shape edge represents activation and a flat-head edge means inhibition. Pink nodes denote the proteins that have experimental measurements. The dash line denotes the predicted drug effects, *i.e.*, signal transduction is blocked here.

For each of the 32 signaling proteins [11], we simulated the knock-down and ranked them according to the RMSE. STAT3, p27, p53, ERK and HSP27 are the top 5 proteins with the smallest RMSE. Therefore, we assumed that the drug target should be the common upstream node of these top-ranked proteins. Next we extracted pathway information from the GeneGO MetaCore database [32]. Fig 5 displays the pathways that contain the top-ranked proteins as well as some related proteins. We can see that EGFR is the common upstream node of all the 5 proteins thus it is inferred as the drug target. A red edge denotes that the reaction should be removed from the pathway, since the signal transduction from EGFR to ERK is blocked. In [11], the drug (erlotinib) used is indeed known as an EGFR inhibitor. Erlotinib is a small molecule that is able to block the signal transduction downstream of EGFR, such as the PI3K-AKT or the RAS-RAF-MAPK-MEK-ERK pathway [33]. In our experiment, however, AKT was ranked the fifth from the bottom, meaning the signals transmitted to AKT were not blocked, which suggests that the PI3K-AKT pathway in Fig 5 cannot be removed although the drug targets EGFR. This interpretation was also consistent with that in [30], the authors of which identified erlotinib-induced pathway alterations using Integer Linear Programming and predicted that the RAS-RAF-MAPK-MEK-ERK cascade was removed while the PI3K-AKT network was retained. This is because the PI3K-AKT pathway was used by other pathways which cannot be blocked by erlotinib. Therefore, knowing that the drug is well designed to hit certain molecules is not sufficient for identifying the drug effects. The experiment shows that the proposed model is able to identify not only the drug target but also the drug effects by rewiring signaling pathways *in silico*, which shows a great potential to complement the analysis based on the drug’s biochemical activity (*e.g.*, binding affinities). In addition, the *in silico* simulation of protein knock-down was executed using the linear model to test if the linear model is able to identify the drug targets and drug effects. Following the same process aforementioned, the top 5 proteins with the relatively small RMSE between the linear model predictions and the real data were Caspase 9, BID, RIP, JNK and S6. However, based on GeneGO MetaCore database [32], Caspase 9, BID, JNK and S6 are all downstream of PI3K-AKT pathway which should not be blocked by an EGFR inhibitor since PI3K-AKT pathway is used by other pathways (*e.g.*, TNFR-PI3K-AKT) [30]. There was no evidence of RIP being downstream of EGFR [32]. Therefore, the linear model showed limited capability of identifying drug targets and drug effects.

#### Identifying time-staggered input-output relationship

**Fig 6:**
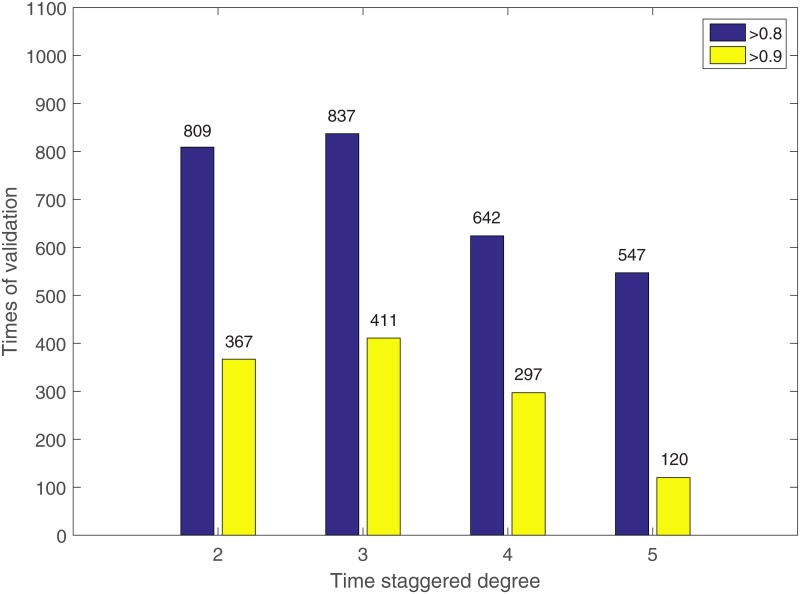
Performance of the linear and the nonlinear models at different time-staggered degrees. Blue bars and yellow bars indicate the numbers of validations which have both Spearman and Pearson correlation coefficients higher than 0.8 and 0.9, respectively.

The process of phosphorylation of signaling proteins and the transduction of the signals to the downstream pathways are accomplished within minutes. On the other hand, it may take hours for the cells to adjust its phenotypes in response to the input signals. Therefore, the identification of time-staggered input-output relationships is very important. We extracted 10 time points (*i.e.*, 0, 0.1, 0.25, 0.5, 1, 2, 4, 6, 8 and 12 hours) from the data set in [11]. The phosphoproteomics data cover time points form 0 to 7 and the cell fate data are measured at time points 0 and from 6 to 9. So we designed experiments (Table 4) to see what time-staggered degree can reveal the most of the input-output relationship (Fig 6). Take the time-staggered degree equal to 2 as an example, we assumed that the “output” at time point *t* is the response to the “input” at time point “*t*−2”. The “output” at time point 0 will be related to the “input” also at time point 0.

**Table 4:** Experimental design for identification of time-staggered input-output relationships.

time-staggered degree	Time Point								
	Input	0	1	2	3	4	5	6	7
2	Output	0				6	7	8	9
3	Output	0			6	7	8	9	
4	Output	0		6	7	8	9		
5	Output	0	6	7	8	9			

We constructed four data sets corresponding to the four time-staggered degrees (Table 4) to predict the cell death. Fig 6 shows the numbers of outputs that have relatively high accuracy. The results generated by the proposed nonlinear model show that it is most reliable that the “output” at time point *t* is the response to the “input” at time point “*t*−3”. The same conclusion can be drawn for the linear model.

#### Cell line discrimination

**Fig 7:**
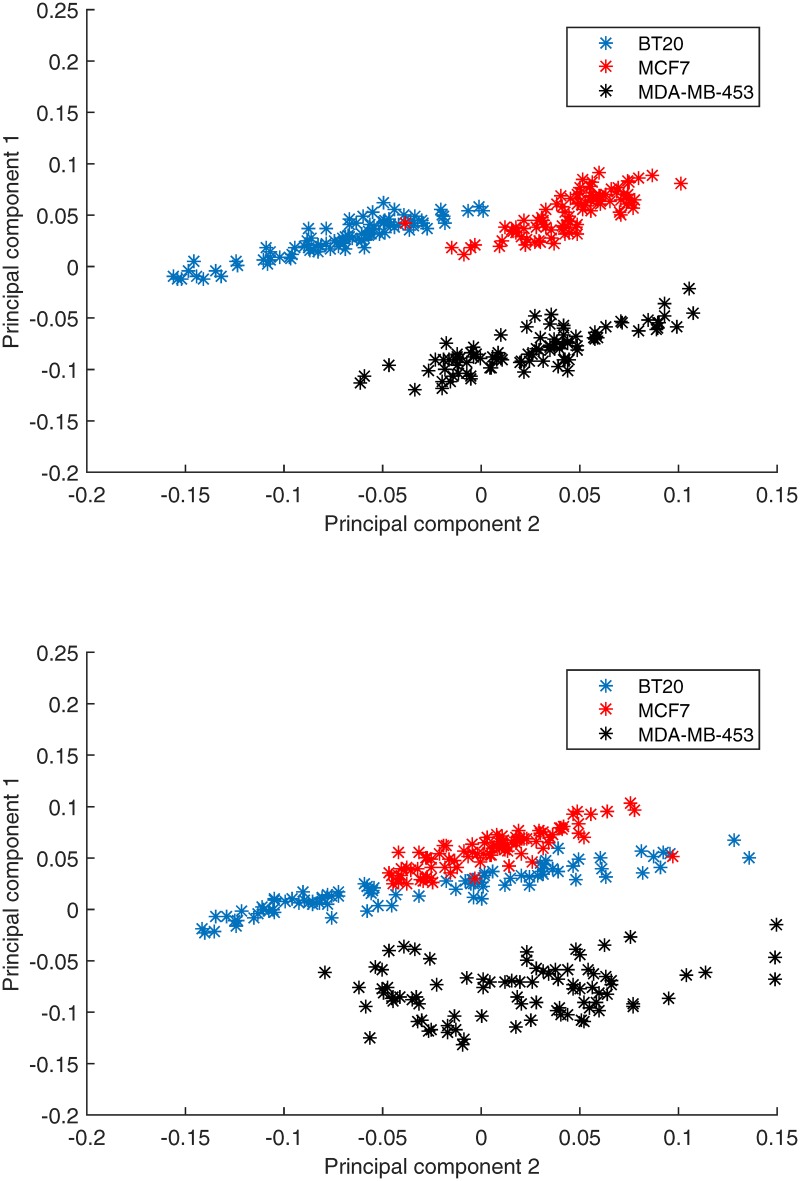
Principal component analysis for cell line discrimination. (a) The nonlinear model. (b) The linear model.

To model a biological system statistically, it is important to capture the common features while keeping the specific characteristics between different cell lines. Therefore, we designed an experiment to verify the performance of our nonlinear model in cell line discrimination. Signaling and apoptosis data from all the three cell lines [11] were combined together. Principal components were extracted using PLSR (for the nonlinear model, PLSR was used after log transformation) and observations of signaling proteins were projected against the first two principal components (Fig 7(a)). Blue, red and black asterisks represent observations from the BT20, MCF7 and MDA-MB-453 cell lines, respectively. The same process was done using the linear model and the result is shown in Fig 7(b). It is clear that the observations are highly cell line dependent. BT20 (red cluster) and MCF7 (green cluster) have similar features on the first principal component while MDA-MB-453 has a significantly different behavior. On the other hand, the second principal component captures variance that can be used to distinguish BT20 and MDA-MB-453 from MCF7. Based on the linear model, a significant overlap can be found between the MCF7 and MDA-MB-453 clusters and the BT20 cluster is slightly scattered. By contrast, the 3 clusters generated by our nonlinear model are more separated and concentrated. Specifically, over ten data points are misclassified (*e.g.*, an observation from MCF7 is classified into the BT20 cluster) by the linear model while there is only one misclassified data point by the nonlinear model.

### Results on HT-29 cell line

In the above experiments, the activity level of each signaling protein is considered as an independent variable and the dimension of the independent variables is the number of signaling proteins. For HT-29 cell line data, we constructed a relatively high dimensional space for independent variables [9, 10] besides the dimensions of the signaling proteins. In particular, the independent variables comprise the 19 signals at all 13 time points (247 independent variables), the instantaneous-derivative between each pair of adjacent time points for all the 19 proteins (247 independent variables), the maximum signal, the mean signal and the steady-state signal for each protein, etc. All together, a 570-dimensional space was constructed and the top 20 most informative dimensions identified in [9] were all included. For dependent variables, a 12-dimensional space was extracted (4 types of cellular responses at 3 time points) and all the data were normalized into the interval (0, 1) using the sigmoid function.

We then compared the performance of the model proposed in [10] and our proposed model in the prediction of cell death using a leave-one-out cross-validation. RMSE between the model predictions and the testing data are 2.11 and 1.77 for the model of Gaudet *et al.* [10] and our model, respectively. Our model achieves a lower RMSE, indicating that it could better predict the cell apoptotic responses.

**Fig 8:**
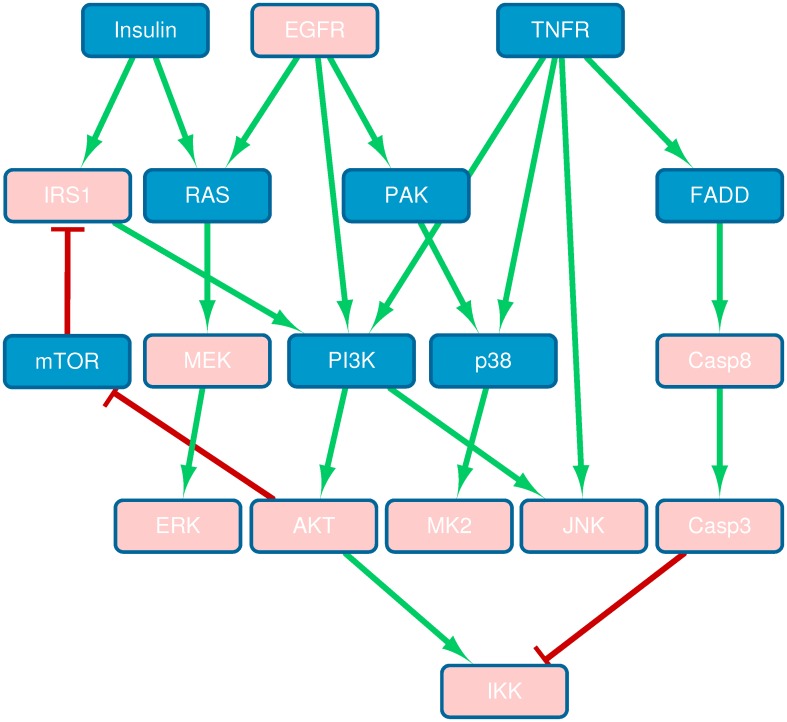
Schematic representation of the signaling network induced by TNF, EGF, and insulin [10, 32]. The measured signals are highlighted in pink. Green lines with arrows and red lines with flat-heads indicate activation and inhibition, respectively.

We also designed experiments following the same procedure as in Section “Drug effects identification by *in silico* simulation of protein knock-down” to identify the effect of the perturbations on the pathways. Caspase-3, which plays a crucial role in the execution-phase of cell apoptosis, was selected as the dependent variable. All the remaining 18 proteins were treated as independent variables. The control group of the dataset in [10] was used for training the parameters in Eqs (4) and (7). The observations treated by one of the 3 stimuli (*i.e.*, TNFR, EGFR and Insulin) were first extracted as the perturbed group. The *in silico* protein knock-down was simulated and the proteins which induce relative large discrepancy with the real data after knock-down are identified as the enhanced signals by the stimuli. The typical targets of the stimuli are all ranked very high as expected, such as JNK and IKK (ranked 3rd and 4th) for TNFR; EGFR and AKT (1st and 3rd) for EGFR; ERK, MEK and AKT (1st, 4th and 5th) for Insulin [10]. However, when we looked into the combination of TNFR and EGFR, the signal flows of TNFR-JNK and TNFR-IKK were significantly weakened (11th and 18th), and the signal of AKT was enhanced (2nd). On the other hand, significant reduction of the AKT signal (ranked 16th) was detected under the the combination of TNFR and Insulin. This suggests that the synergistic effect of TNFR and EGFR could enhance the signal flow PI3K-AKT, while the cascades of TNFR-PI3K-AKT and Insulin-IRS1-PI3K-AKT could conflict with each other when both TNFR and Insulin are present (Fig 8).

### Results on DREAM8 dataset

The DREAM8 data released in 2013 are normalized using a procedure developed in [34]. We reversed the procedure to generate the raw data as follows. First, we multiplied each normalized linear value by the correction factor to get the median-centered ratio in linear value. Second, we converted median-centered ratios in a linear value to a median-centered *log*_2_ value. Then, we divided each median-centered data by its standard deviation for normalization. Since no cell response were directly measured in the data set, we iteratively selected one signaling protein as the dependent variable and the remaining signaling proteins were regarded as independent variables.

Within each cell line, the dataset was divided into two subsets, *i.e.*, two thirds were used for training and one third for testing. On the training part, leave-one-out cross-validation was employed to learn the parameters in Eq (4). With the mean values of the parameters from the cross-validation, we were able to make predictions on the testing data. We repeated the above procedure for 100 times. Table 5 shows the RMSE which estimates the accuracy of the model predictions for each cell line. It is clear that our nonlinear model predicts the biological system with a higher accuracy than the linear model.

**Table 5:** Performance of the nonlinear and linear model on DREAM8 dataset. The values are the RMSE between the model predictions and testing data of activity of signaling proteins.

Cell lines	Nonlinear model	Linear model
BT20	0.41	0.71
BT549	0.46	0.69
MCF7	0.33	0.39
UACC812	0.57	1.46

## Conclusions

Data-driven models are able to provide new biological insights by analysing a dataset itself. They are particularly useful when the underlying molecular mechanisms are unclear. In this paper, we proposed a nonlinear power-law model to describe the relationship between cell fates and cell signals. Simulations based on an extended model of Boolean network and ordinary differential equations (ODEs) provideed hints about the form of the nonlinear function as well as how to validate the proposed model. By predicting the cell responses, we compared the performance of our nonlinear model with the linear model on 3 real data sets, demonstrating that the proposed nonlinear model has higher accuracy than the linear model. Given network topology, the proposed model performs well on drug target identification and is able to reveal the drug effects by rewiring the signaling pathways *in silico*. Then our nonlinear model was used for time-staggered input-output relationships identification and cell line discrimination.

In spite of the promising performance of our proposed model, limitations have also been noticed. First, as a data-driven method, it is unable to incorporate prior biological knowledge to take into account the underlying mechanisms. Second, our model has not been tested for the situation when more than one signaling proteins are inhibited or the corresponding genes are knocked out at the same time. Moreover, when we compared the performance of the linear and the nonlinear models in predicting cell fate across cell lines, the superiority of the proposed model may not significant enough. In future, we will include network topology to improve the model so that it can deal with synergistic effect of multiple perturbations.

## Supporting Information

S1 File SimBoolNet data. The compressed zip file contains the data in ‘.mat’ format which can be accessed by MATLAB. This data file comprises the outputs of SimBoolNet when simulated the dynamics of the network in Fig 1(a). The rows and the columns of the data matrix represent the nodes in Fig 1(a) (*i.e.*, Casp8, Casp9, OncogenicSignature, Casp3, CellDeath, DNADamage and EGFR) and the simulation iterations. Besides the simulation described in the main text, another 10 times of simulations using different parameter settings are provided to explore the robustness of the curve fitting results to the parameters.
(ZIP)

S2 File CellDesigner data. The compressed zip file contains the ‘.mat’ data which comprises the outputs of CellDesigner when simulated the dynamics of the network in [9]. The rows and the columns of the data matrix represent the simulation iterations and the 112 species in the ODE model of [9].
(ZIP)

S3 File Breast cancer cell lines data. The compressed zip file contains the ‘.mat’ data which is from the published work of Lee *et al.* [11]. The data are separated into 3 data files according to the breast cancer cell lines, *i.e.* BT20, MDA-MB-453 and MCF7. Each data file comprises the measurements of signaling proteins and cell fates from the same cell line. The rows and the columns of the data matrix represent the observations and the signaling proteins (or cell fates), respectively.
(ZIP)

S4 File HT-29 cell line data. The compressed zip file contains the ‘.mat’ data which is from the published work of [10]. The rows and the columns of the data matrix represent the observations and the signals (or cell responses), respectively.
(ZIP)

S5 File DREAM8 data. The compressed zip file contains the ‘.mat’ data which is from the DREAM8 challenge in 2013 [17]. There are four cell lines, namely BT20, BT549, MCF7 and UACC812. The rows and the columns of the data matrix represent the observations and the signals, respectively.
(ZIP)

## References

[1] Calzone L, Tournier L, Fourquet S, Thieffry D, Zhivotovsky B, Barillot E, Zinovyev A. Mathematical modelling of cell-fate decision in response to death receptor engagement. PLoS Comput Biol. 2010 3 5; 6(3):e1000702 10.1371/journal.pcbi.1000702 20221256 PMC2832675

[2] Leontaritis IJ, Billings SA. Input-output parametric models for nonlinear systems. Part I: deterministic non-linear systems. International Journal of Control. 1985; 41(2):303–328. 10.1080/0020718508961129

[3] Leontaritis IJ, Billings SA. Input-output parametric models for non-linear systems. Part II: stochastic non-linear systems. International Journal of Control. 1985; 41(2):329–344. 10.1080/0020718508961130

[4] Newman MEJ. Power laws, Pareto distributions and Zipf’s law. 2004 12; 46(5):323–351.

[5] Clauset A, Shalizi CR, Newman MEJ. Power-Law Distributions in Empirical Data. SIAM Rev. 2009 2; 51(4):661–703. 10.1137/070710111

[6] Lu ET, Hamilton RJ. Avalanches of the distribution of solar flares. 1991 11; 380(2):89–92.

[7] Zanette DH, Manrubia SC. Vertical transmission of culture and the distribution of family names. 2001 6; 295(1-2):1–8.

[8] Kleiber M. Body size and metabolism. Hilgardia. 1932; 6:315–351. 10.3733/hilg.v06n11p315

[9] Janes KA, Albeck JG, Gaudet S, Sorger PK, Lauffenburger DA, Yaffe MB. A systems model of signaling identifies a molecular basis set for cytokine-induced apoptosis. Science. 2005 12 9; 310(5754):1646–53. 10.1126/science.1116598 16339439

[10] Gaudet S, Janes KA, Albeck JG, Pace EA, Lauffenburger DA, Sorger PK. A compendium of signals and responses triggered by prodeath and prosurvival cytokines. Mol Cell Proteomics. 2005 10;4(10):1569–90. Epub 2005 Jul 18. 10.1074/mcp.M500158-MCP200 16030008

[11] Lee MJ, Ye AS, Gardino AK, Heijink AM, Sorger PK, MacBeath G, Yaffe MB. Sequential application of anticancer drugs enhances cell death by rewiring apoptotic signaling networks. Cell. 2012 5 11; 149(4):780–94. 10.1016/j.cell.2012.03.031 22579283 PMC3501264

[12] Bullinger E. System Analysis of a Programmed Cell Death Model. Proc. of the 44th IEEE Conf. on Decision and Control and European Control Conference, ECC, Seville, Spain. 2005:7994-7999.

[13] Higgins JP. Nonlinear systems in medicine. The Yale Journal of Biology and Medicine. 2002; 75(5-6): 247–260. 14580107 PMC2588816

[14] Legewie S, Bluthgen N, Herzel H. Mathematical modeling identifies inhibitors of apoptosis as mediators of positive feedback and bistability. PLoS Comput Biol. 2006 9 15; 2(9):e120 Epub 2006 Jul 28. 10.1371/journal.pcbi.0020120 16978046 PMC1570177

[15] Eissing T, Conzelmann H, Gilles ED, Allgöwer F, Bullinger E, Scheurich P. Bistability analyses of a caspase activation model for receptor-induced apoptosis. J Biol Chem. 2004 8 27; 279(35):36892–7. Epub 2004 Jun 18. 15208304 10.1074/jbc.M404893200

[16] Callard R, George AJ, Stark J. Cytokines, chaos, and complexity. Immunity. 1999 11; 11(5):507–13. 10.1016/S1074-7613(00)80125-9 10591175

[17] HPN-DREAM breast cancer network inference challenge. Available: http://dreamchallenges.org/.

[18] Janes KA, Yaffe MB. Data-driven modelling of signal-transduction networks. Nat Rev Mol Cell Biol. 2006 11; 7(11):820–8. 10.1038/nrm2041 17057752

[19] Zheng J, Zhang D, Przytycki PF, Zielinski R, Capala J, Przytycka TM. SimBoolNet—a Cytoscape plugin for dynamic simulation of signaling networks. Bioinformatics. 2010 1 1; 26(1):141–2. Epub 2009 Nov 3. 10.1093/bioinformatics/btp617 19887508 PMC2796819

[20] Savageau MA. Biochemical systems analysis. I. Some mathematical properties of the rate law for the component enzymatic enzymatic reactions. J Theor Biol. 1969 12; 25(3):365–369. 10.1016/S0022-5193(69)80026-3 5387046

[21] Savageau MA. Biochemical systems analysis. II. The steady-state solutions for an n-pool system using a power-law approximation. J Theor Biol. 1969 12; 25(3):370–379. 10.1016/S0022-5193(69)80027-5 5387047

[22] Bode HW. Network Analysis and Feedback Amplifier Design. Princeton, NJ: Van Nostrand; 1945.

[23] Novák B, Tyson JJ. A model for restriction point control of the mammalian cell cycle. J Theor Biol. 2004 10 21; 230(4):563–79. 10.1016/j.jtbi.2004.04.039 15363676

[24] Tian T, Smith-Miles K. Mathematical modeling of GATA-switching for regulating the differentiation of hematopoietic stem cell. BMC Syst Biol. 2014; 8 Suppl 1:S8 Epub 2014 Jan 24. 10.1186/1752-0509-8-S1-S8 24565335 PMC4080254

[25] Dunster JL, Mazet F, Fry MJ, Gibbins JM, Tindall MJ. Regulation of Early Steps of GPVI Signal Transduction by Phosphatases: A Systems Biology Approach. PLoS Comput Biol. 2015 11 19; 11(11):e1004589 eCollection 2015. 10.1371/journal.pcbi.1004589 26584182 PMC4652868

[26] Prescott TP, Lang M, Papachristodoulou A. Quantification of Interactions between Dynamic Cellular Network Functionalities by Cascaded Layering. PLoS Comput Biol. 2015 5 1; 11(5):e1004235 eCollection 2015. 10.1371/journal.pcbi.1004235 25933116 PMC4416712

[27] Funahashi A, Tanimura N, Morohashi M, Kitano H. CellDesigner: A process diagram editor for gene-regulatory and biochemical networks. BIOSILICO. 2003 11; 1(5). 10.1016/S1478-5382(03)02370-9

[28] Li C, Donizelli M, Rodriguez N, Dharuri H, Endler L, Chelliah V, Li L, He E, Henry A, Stefan MI, Snoep JL, Hucka M, Le Nov ère N, Laibe C. BioModels Database: An enhanced, curated and annotated resource for published quantitative kinetic models. BMC Syst Biol. 2010 6 29;4:92 10.1186/1752-0509-4-92 20587024 PMC2909940

[29] Kullback S, Leibler RA. On Information and Sufficiency. The Annals of Mathematical Statistics. 1951 3; 22:79–86.

[30] Mitsos A, Melas IN, Siminelakis P, Chairakaki AD, Saez-Rodriguez J, Alexopoulos LG. Identifying drug effects via pathway alterations using an integer linear programming optimization formulation on phosphoproteomic data. PLoS Comput Biol. 2009 12;5(12):e1000591 Epub 2009 Dec 4. 10.1371/journal.pcbi.1000591 19997482 PMC2776985

[31] Zhang F, Wu M, Li XJ, Li XL, Kwoh CK, Zheng J. Predicting essential genes and synthetic lethality via influence propagation in signaling pathways of cancer cell fates. J Bioinform Comput Biol. 2015 6;13(3):1541002 Epub 2015 Jan 11. 10.1142/S0219720015410024 25669329

[32] Ekins S, Nikolsky Y, Bugrim A, Kirillov E, Nikolskaya T. Pathway mapping tools for analysis of high content data. Methods Mol Biol. 2007;356:319–50. 16988414 10.1385/1-59745-217-3:319

[33] Boeck S, Jung A, Laubender RP, Neumann J, Egg R, Goritschan C, Vehling-Kaiser U, Winkelmann C, Fischer von Weikersthal L, Clemens MR, Gauler TC, Märten A, Klein S, Kojouharoff G, Barner M, Geissler M, Greten TF, Mansmann U, Kirchner T, Heinemann V. EGFR pathway biomarkers in erlotinib-treated patients with advanced pancreatic cancer: translational results from the randomised, crossover phase 3 trial AIO-PK0104. Br J Cancer. 2013 2 5;108(2):469–76. Epub 2012 Nov 20. 10.1038/bjc.2012.495 23169292 PMC3566829

[34] MDAnderson RPPA resource. Available: https://www.mdanderson.org/education-and-research/resources-for-professionals/scientific-resources/core-facilities-and-services/functional-proteomics-rppa-core/faq/functional-proteomics-reverse-phase-protein-array-core-facility-faq.html

